# Optimization of deficit irrigation system for drip-irrigated corn in northern Xinjiang using dynamic reconstruction and dual physics-informed neural networks to drive AquaCrop

**DOI:** 10.3389/fpls.2025.1678277

**Published:** 2025-09-19

**Authors:** Haonan Zhang, Jinghua Zhao, Ming Hong, Liang Ma

**Affiliations:** ^1^ College of Hydraulic and Civil Engineering, Xinjiang Agricultural University, Urumqi, Xinjiang, China; ^2^ Xinjiang Key Laboratory of Water Engineering Safety and Water Disaster Prevention, Urumqi, Xinjiang, China

**Keywords:** corn, deficit irrigation, AquaCrop model, optimization method, DPINNs

## Abstract

**Introduction:**

To optimize the irrigation schedule for corn in northern Xinjiang and save water resources while maintaining stable production.

**Methods:**

Based on the actual water shortage in northern Xinjiang during summer 2024, this study set up different deficit irrigation gradient treatments according to the crop water requirement (ET_c_) of each growth stage of corn. Combined with the corn growth and yield data of farmers from 2022 to 2024, the model parameters were calibrated and validated through global sensitivity analysis using AquaCrop-OS MATLAB. Then, the Dynamic Reconstruction and Dual Physics-Informed Neural Networks (DR-DPINNs) were integrated with water balance constraints during the corn growth period to optimize the deficit irrigation system for corn in northern Xinjiang.

**Results:**

The results showed that in the global sensitivity analysis of the AquaCrop model, the water productivity (wp) and canopy growth coefficient (cgc) parameters had a significant impact on biomass accumulation (STi>0.10), and the canopy senescence parameter (psen) had a marked effect on yield (Si>0.05). The model parameters obtained through sensitivity analysis could meet the application requirements for simulating biomass, canopy cover, soil water content, and yield in the AquaCrop model. After optimization with DR-DPINNs, when the total irrigation amount was 472 mm, the yield increased by 10.8% and the water use efficiency rose by 11.15% compared with the conventional scheme. The DR-DPINNs method, by combining physical mechanisms with dynamic feature extraction, could significantly enhance the solving capability for high-dimensional nonlinear irrigation optimization problems. The optimized spatial and temporal irrigation distribution under a total water volume of 472 mm could achieve a simultaneous increase in yield and water use efficiency.

**Discussion:**

This study can provide theoretical methods with both mechanistic interpretability and decision-making accuracy for the dynamic optimal systems of drip-irrigated corn under water resource constraints in arid regions, and offer theoretical support and technical reference for agricultural water management in arid regions.

## 1 Introduction

Maize is one of the most widely grown and high-yielding crops globally and plays a vital role in food supply, energy production, feed manufacturing, and industrial applications (Hammad et al., 2018; Erenstein et al., 2022). In northern Xinjiang, China, located in an inland arid zone with scant precipitation and intense evaporation, agriculture relies heavily on irrigation. Here, water resource shortages and uneven distribution severely restrict maize production (Shen et al., 2013).

Water is not only essential for maize’s physiological and biochemical processes but also indirectly affects yield formation by influencing root activity, nutrient uptake, and the allocation of photosynthetic products (Du et al., 2013; Pettigrew, 2016; Zhang et al., 2023) **Tables 1** and **2**. During its growth, maize is subject to multiple factors: fluctuating meteorological elements, soil moisture heterogeneity, and crop-environment interactions. During its growth, maize is subject to multiple factors: fluctuating meteorological elements, soil moisture heterogeneity, and crop-environment interactions. These factors are linked by complex nonlinear relationships and exhibition evolution characteristics (Zhou et al., 2020; Chen et al., 2021). Deficit irrigation, a strategy to balance water conservation and stable production by regulating water supply, shows great potential for alleviating water resource conflicts. Yet, its precise implementation hinges on a deep understanding of crop-water dynamics and reliable model predictions (Ahmadpour et al., 2022; Geerts and Raes, 2009). The AquaCrop model stands out for its mechanistic approach to crop-water relationships and relatively low data requirements, making it widely used in irrigation strategy evaluations. However, it falls short in extreme water scarcity conditions and regional parameter optimization (Fidencio Cruz-Bautista et al., 2023; Ghazouan et al., 2019). Traditional static models also struggle to capture the dynamic responses of complex environment-crop-soil systems, especially their spatiotemporal variations. They have difficulties in accurately quantifying the cascading effects of water deficits on crop growth, yield formation, and water use efficiency across different growth stages, leading to lagging and empirical irrigation strategy optimization (Tolomio and Casa, 2020). In the context of sustainable agricultural development, enhancing the precision of irrigation management, understanding the water requirements of maize at different growth stages, and developing precise irrigation decision models based on physiological and ecological processes to achieve efficient water use and increased yields remain key challenges in agricultural water resource management.

**Table 1:** Deficit irrigation treatments in 2024.

Year	Number	Stage specific irrigation management			
		Initial growth stage	Rapid growth stage	Mid growth stage	Late growth stage
2024	CK	NC	100%ET_c_	100%ET_c_	NC
	T1	NC	80%ET_c_	100%ET_c_	NC
	T2	NC	60%ET_c_	100%ET_c_	NC
	T3	NC	100%ET_c_	80%ET_c_	NC
	T4	NC	100%ET_c_	60%ET_c_	NC
	T5	NC	80%ET_c_	80%ET_c_	NC
	T6	NC	80%ET_c_	60%ET_c_	NC
	T7	NC	60%ET_c_	80%ET_c_	NC
	T8	NC	60%ET_c_	60%ET_c_	NC

NC stands for equal amount of non-regulated irrigation. The same applies to the following table.

Physics-Informed Neural Networks (PINNs) can simulate complex nonlinear processes using fully connected neural networks while incorporating physical information constraints. This ensures that the neural network approximates the solution of the physical system and aligns its outputs with physical laws, thus avoiding the “black box” problem (Li, 2025; Guo et al., 2023). This approach effectively combines mechanistic models with data-driven methods, enhancing the interpretability and predictive accuracy for complex nonlinear systems. It also improves the precision and reliability of irrigation decisions (Oikawa and Saito, 2024). In studies integrating crop models with data-driven approaches, the high-dimensional parameter space of AquaCrop outputs can represent the mechanisms of crop-soil-atmosphere interactions. However, direct coupling with PINNs often leads to dimensionality and parameter conflicts (Oulaid et al., 2024). Research shows that high-dimensional outputs dramatically increase the number of nodes in the neural network input layer. This not only increases model complexity and computational burden but also causes multicollinearity and overfitting risks in parameter optimization due to redundant features (Advani et al., 2020). Dynamic Reconstruction (DR), on the other hand, can reduce model complexity by performing dimensionality reduction and feature extraction on high-dimensional and complex data while retaining key system characteristics (Mainali et al., 2021). Studies have demonstrated that DR can map the high-dimensional parameter space of outputs to low-dimensional intrinsic dynamic patterns through dimensionality reduction and feature extraction. This approach preserves the physical core of mechanistic models and provides structured inputs for neural networks (Lumbut and Ponnoprat, 2024).

Previous studies have made progress in integrating process-based crop models with data-driven methods. However, further research is needed on irrigation optimization across multidimensional scales and dynamic scenarios, particularly on dynamically unraveling the water response mechanisms of maize under multi-stage compounded deficit irrigation. Although the AquaCrop model is one of the most commonly used tools for simulating maize growth dynamics, few studies have explored the coupling mechanisms of non-steady-state irrigation and crop stage-specific sensitivity responses for irrigation optimization. Understanding the compounded effects of water deficits and stage-specific sensitivity differences in maize growth stages is crucial for constructing precision irrigation decision models based on crop mechanistic processes. This study focuses on drip-irrigated maize in Xinjiang. It integrates DR with Double Physics-Informed Neural Networks (DR-DPINNs) to establish a dual-driven framework for optimizing irrigation systems based on process and data. According to the actual water shortage in northern Xinjiang in summer 2024, different water deficit irrigation treatments were set up in 2024 based on the water requirements of each growth stage of maize. This was combined with the growth and yield data of maize from farmer practice from 2022 to 2024.Global sensitivity analysis was carried out using AquaCrop-OS MATLAB to screen key parameters and complete regional adaptive calibration. Specifically, we utilized DR to map the high-dimensional parameter space of AquaCrop outputs to a low-dimensional intrinsic dynamic space. This allowed us to extract dominant feature modes with explicit agronomic meanings. These modes served as the input basis for the double neural network. Furthermore, we constructed a physically constrained irrigation optimization method by coupling soil water balance equations with stage-sensitive crop water production functions. This method comprehensively analyzed the effects of water deficit thresholds on yield and water use efficiency across different maize growth stages. Ultimately, it clarified a dynamic irrigation scheduling system suitable for drip-irrigated maize under water resource constraints in northern Xinjiang. This study offers theoretical support and technical guidance for agricultural water management in arid regions.

## 2 Materials and methods

### 2.1 Physical overview of the experimental area

The experiment was conducted from April to September 2024 at the Agricultural Integrated Development Area in Karamay, Xinjiang Uygur Autonomous Region, China (85°33′E, 45°46′N, elevation 270.00 m). The area has a typical continental arid desert climate: mean annual temperature, 8.1 °C; annual radiation, 5430–6670 MJ/cm²; mean annual wind speed, 3.7 m/s; mean annual precipitation, 101.1 mm; mean annual evaporation, 3545.2 mm; annual sunshine duration, 2600–3400 h; frost-free period, 197–268 days. The soil has a mean dry bulk density of 1.47 g/cm³, a field water capacity (volumetric) of 36.07%, and a groundwater depth of 2–4 m. Before maize planting, the fertility of the topsoil (0–20 cm) was: pH 8.32, soil organic matter 17.69 g/kg, alkaline nitrogen 59.82 mg/kg, available phosphorus 13.55 mg/kg, and available potassium 135.32 mg/kg.

### 2.2 Experimental materials and experimental design

This study sampled data from farmer-managed drip-irrigated maize fields in Karamay for three consecutive years from 2022 to 2024 and conducted a deficit irrigation experiment during different maize growth stages in 2024, using the Xinnong 008 variety. The sowing date was April 30, with the harvest date being September 13 in 2022; the sowing date was May 30, with the harvest date being September 13 in 2023; and the sowing date was April 25, with the harvest date being September 2 in 2024. An alternating wide-narrow row planting pattern was used. The drip tape spacing was 1.1 m, the narrow row spacing was 0.4 m, the wide row spacing was 0.7 m, and the plant spacing within the row was 0.2 m. The dripper spacing was 25 cm, with a flow rate of 2.8 L/h and a rated working pressure of 0.1 MPa. The seeding density was 80, 000 plants per hectare. The deficit irrigation experiment set three irrigation levels: 60%, 80%, and 100% of crop evapotranspiration (ET_c_). Based on past field trials in northern Xinjiang, water shortages mainly occur in early and mid-summer. The growth stages were defined using the FAO-56 method (Allen D. G. et al., 1998): early growth (sowing to jointing), rapid growth (jointing to tasseling), mid-growth (tasseling to milk stage), and late growth (milk stage to harvest). Deficit Irrigation Treatments in 2024 and Farmer Control Irrigation Treatments for Corn from 2022 to 2024 are presented in **Tables 1** and **2**, respectively. Each experimental group had two randomized block repeats, totaling three repeats. There were nine main-factor experimental plots and 27 secondary plots. Each main plot was 4 m × 6 m × 3 = 72 m^2^, with a total experimental area of 72 m^2^ × 9 = 648 m^2^. During the growing period, irrigation was carried out every 7 days for a total of 12 times, and nitrogen (urea) at 500 kg/ha, phosphorus (monopotassium phosphate) at 180 kg/ha, and potassium (potassium sulfate) at 135 kg/ha were applied through drip irrigation without basal fertilizer. The fertilizer was applied in three stages: 30% at jointing, 30% at tasseling, and 40% at grain filling.

**Table 2:** Farmer control irrigation treatments for corn from 2022 to 2024.

Year	Number	Stage specific irrigation management			
		Initial growth stage	Rapid growth stage	Mid growth stage	Late growth stage
2022	QC	NC	NC	NC	NC
2023	QC	NC	NC	NC	NC
2024	QC	NC	NC	NC	NC

QC stands for the farmers’ control irrigation.

### 2.3 Measurement of leaf area index and canopy coverage

Measure soil moisture before and after each irrigation. Take the 7th fully expanded leaf from the top before tasseling, and ear leaves after tasseling. Measure chlorophyll content at the leaf tip, center, and base, and average the values. Calculate leaf area index (LAI) using Equation 1 and canopy cover (CC) using Equation 2 (Hsiao et al., 2009).


(1) $$LAI=\frac{\frac{1}{m}\sum_{i=1}^{N}L_{i}\times D_{i}\times K\times D_{r}}{S}$$

(2) $$CC=1.005{\left[1-EXP(-0.6LAI)\right]}^{1.2}$$

In the formula, *LAI* is the leaf area index; *m* is the number of sampled plants; *n* is the total number of leaves on the sampled plants; *L_i_* is the leaf length in cm; *D_i_* is the leaf width in cm; *K* is the leaf area correction coefficient, taken as 0.75; *D_r_* is the plant density in plants per m²; *S* = 10000 cm²/m².

### 2.4 Determination of dry matter accumulation in maize

Before and after each irrigation, the dry matter accumulation of corn was measured using the destructive sampling method. Three uniformly growing plants were selected, and the stems, roots, leaves, and ears of the corn were separately weighed for dry weight. For dry weight measurement, samples were killed green at 105°C for 30 minutes, then dried at 70°C until a constant weight was achieved before being weighed.

### 2.5 Determination of soil water content and crop water consumption

At each irrigation, soil samples from 0–100 cm depth were taken to measure soil water content by drying. Crop water consumption was calculated from pre - and post - irrigation soil water content measurements, accounting for irrigation, precipitation, and percolation.

### 2.6 Measurement of maize yield and water use efficiency

At harvest, 10 representative maize plants from the middle of each plot were manually selected, with three replicates. Within each replicate, a 1 m² area was randomly selected for sampling. After threshing, the fresh weight was measured using an electronic balance with an accuracy of 0.01 g. The samples were then dried in an oven at 60°C until a constant weight was achieved and weighed again. The data obtained from each plot were averaged to calculate the yield per unit area.

he calculation of water consumption employed Equation 3, obtained through the water balance method:


(3) ET=P+I−ΔSWS−R−D

where *ET* is the crop water consumption, P is the rainfall during the maize growing period, *I* is the total irrigation amount during the maize growing period, *ΔSWS* is the soil water storage change, *R* is the surface runoff, and *D* is the deep percolation of soil water. The units of all parameters are mm. Since the water flux changes are very small under the experimental conditions, surface runoff and deep percolation are not considered.

Water use efficiency (WUE) is calculated using Equation 4:


(4) $$WUE=\frac{Y}{ET}$$

where *WUE* is the water use efficiency (kg hm^-2^ mm^-1^), and *Y* is the maize yield per unit area (kg hm^−2^).

### 2.7 Meteorological conditions

**Figure 1:**
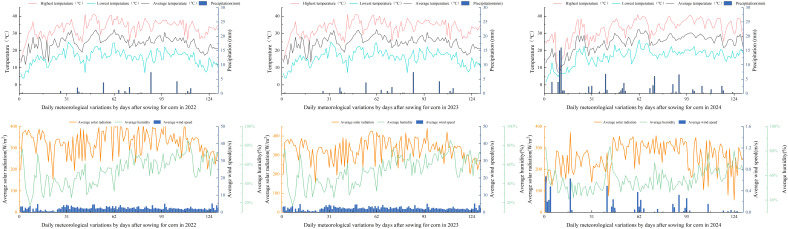
Daily variation of rainfall and average temperature during the maize growing season (2022–2024).

The weather station at the experimental site monitored daily average temperature (Tavg, °C), maximum temperature (Tmax, °C), minimum temperature (Tmin, °C), precipitation (P, mm), sunshine duration (SSD, h), wind speed at 2 m (W, m/s), and relative humidity (RHU, %). The reference evapotranspiration (ET_0_) for the model was calculated using the Penman-Monteith equation from the FAO Irrigation and Drainage Paper 56 (Allen R. at al., 1998). **Figure 1** shows the precipitation (P) and mean temperature (Tavg) during the growing period.

### 2.8 AquaCrop model database

The AquaCrop model, a typical water - driven model for simulating crop biomass and yield, is widely used in agricultural water management (Steduto et al., 2009). Unlike light - or CO_2_ - driven models, it can predict crop productivity under water stress and rainfed conditions, and simulate yields under various irrigation regimes. Its core equations, evolved from those in FAO’s Irrigation and Drainage Paper 33, estimate crop growth - stage water use and yield based on cumulative water deficits (Doorenbos and Kassam, 1979). We used the AquaCrop - OS - MATLAB version for simulations.

Model performance was evaluated using four metrics: the coefficient of determination (R²), root mean square error (RMSE), Nash - Sutcliffe coefficient (EF), and Willmott’s index of agreement (d).

### 2.9 Data analysis and simulation

This study employs the Extended Fourier Amplitude Sensitivity Test (EFAST) method and the AquaCrop model to analyze and simulate the dry biomass, canopy cover, soil moisture content, and yield of maize in the experimental area. Based on the EFAST method, parameters with a first-order sensitivity index (Si) greater than 0.05 and a total sensitivity index (STi) greater than 0.10 (Si + STi > 0.15) were selected. The parameters were randomly sampled using the Monte Carlo method, with a sample size of 3, 500 (EFAST requires a sample size greater than the number of parameters × 65). Batch calculations (3, 500 × 42 × 3 times) were performed using Matlab, with parallel processing. The results were statistically analyzed, and the crop parameters of the model were determined and validated through sensitivity analysis. After model validation, DR-DPINNs framework was constructed. Latin Hypercube Sampling (LHS) designed AquaCrop model data was used as input for DR-DPINNs. By constructing a loss function with physical constraints, the complex nonlinear relationship between learning and irrigation strategies was captured. This transformed the deficit irrigation optimization problem into a fixed-water-volume optimization task. The irrigation water allocation was dynamically optimized to meet the water requirements of maize, aiming to maximize yield and WUE. Subsequently, the optimized plan was re-input into the AquaCrop model for simulation and comparison with the original plan. The model was simulated using farmer control crop data and yield from 2022 to 2023, validated using farmer control crop data and yield from 2024, and tested with data obtained from the field deficit irrigation experiment conducted in 2024.

### 2.10 PINNs framework construction

PINNs can simulate complex nonlinear processes using fully connected neural networks while incorporating physical constraints. This ensures solutions align with physical laws. In this study, a double-layer, multi-dimensional neural network forms the basis of the deficit irrigation optimization model. The network’s equation is presented as Equation 5:


(5) $$\bar{y^{\left(i\right)}}=f(x^{\left(i\right)},\theta )=\beta g(\omega x^{\left(i\right)}+b)$$

In the equation, $\bar{y^{\left(i\right)}}$ represents the model’s predicted output; *f(x^i^, θ)* enotes the neural network’s representation function; *θ* is the set of neural network parameters; *β*is the weight vector linking the input and hidden layers; *g(τ)=(e^τ^-e^-τ^)/(e^τ^+e^-τ^)* is the tanh activation function of the model; *ω* is the weight matrix connecting the input and hidden layers; and *b* is the bias vector of the hidden layer.

### 2.11 Agricultural soil and water physical constraints

Compared to PINNs, DPINNs excel in capturing nonlinear relationships and multi-scale features in complex physical phenomena. They can automatically learn hierarchical feature representations from input data. In DPINNs, the agricultural soil and water physical constraint loss term (*l_h_*) is calculated by comparing the predicted and actual values of the soil water balance and crop water production functions, derived from partial differential equations ensuring time-continuous conservation. The detailed derivation process is shown in Equation 6:


(6) $$\begin{array}{c} I_{i}+P_{i}+G_{i}=ET_{i}+D_{i}+R_{i}\pm \text{Δ}W_{i}\\ \frac{Y_{a}}{Y_{m}}=\prod_{i=1}^{n}{\left(\frac{ET_{a,i}}{ET_{m,i}}\right)}^{\lambda_{i}} \end{array}\}$$

In the equation, *I_i_*, Pi and *G_i_*, are the soil irrigation amount, rainfall, and groundwater recharge for stage *i*, respectively, ETiis the total evapotranspiration for stage *i*, *D_i_* and *R_i_* are the deep percolation and surface runoff for stage *i*, respectively, *△w_i_* is the change in soil water storage for stage *i*, *Y_a_* is the actual yield, *Y_m_* is the maximum achievable yield under full irrigation conditions, *ET_a, i_* and *ET_m, i_* are the actual and maximum evapotranspiration for stage *i*, respectively, λi is the water stress sensitivity coefficient for stage *i*.

To achieve continuous water balance in time and space, we introduce the vertical profile differential *dz* and the time differential *dt*. After simplifying the differentials, the equation can be expressed as:


(7) $$\begin{array}{c} \frac{\partial \theta (z,t)}{\partial t}=-\frac{\partial q(z,t)}{\partial z}+S(z,t)\\ \frac{Y_{a}}{Y_{m}}=\exp\left[\int_{0}^{T}\lambda (t)f(\theta (t),CC(t))dt\right] \end{array}\}$$

In the equation, *θ(z, t)* represents the soil water content at depth *z*;*q(z, t)* is the water flux in the *z*-direction; *S(z, t)* represents the source and sink term. In solving the equation, $\frac{Y_{a}}{Y_{m}}$ is treated as a small time interval and approximated as a Riemann sum over time *t*.In the limit, this allows $\frac{Y_{a}}{Y_{m}}$ to be simplified for easier explanation:


(8) Y(t)=G(θ(t),CC(t),p)

In the equation, p is a constant that represents all the empirical parameters.

After transforming the original discrete model into a partial differential equation with continuous time conservation form using Equations 7 and 8, the agricultural soil and water physical constraint loss term *l_h_* is represented by the corresponding agricultural soil and water physical constraint residual.


(9) $$l_{h}=\|\frac{\partial \theta_{pred}}{\partial t}+\frac{\partial q(\theta_{pred})}{\partial z}-S(\theta_{pred}){\|}^{2}+\|{\hat{Y}}_{pred}-G(\theta_{pred},CC_{pred}){\|}^{2}$$

### 2.12 Dynamic reconstruction

In agricultural water management and irrigation scheduling optimization, crop growth is influenced by dynamic factors like weather, soil moisture, and growth stages, which have complex nonlinear relationships. Traditional static models often fail to capture these dynamics, leading to low prediction accuracy and limiting irrigation strategy optimization. DR, which reduces dimensionality and extracts features from high-dimensional and complex data, can capture the system’s dynamic evolution, simplify the model, and retain key information. The added physical constraints provide extra information, ensuring reasonable predictions even with limited data, and ensuring the network output aligns with crop production physiology and soil water movement mechanisms, avoiding the “black box” issue. The DR formula is expressed as:


(10) $$u(t)=f_{NN}(x,t;\theta )+D(z(t))$$

In the equation, *u(t)* represents the comprehensive output at time t after integrating dynamic features, fNN is the neural network mapping function, which takes *(x, t)* as its input, In Equation 10, *D(.)* represents the function mapping the reduced - dimension dynamic features *z(t)* to the output space. During training, if the loss in Equation 11 doesn’t converge for a long time, the network adaptively adjusts parameters to enhance the physical equation’s performance. The adaptive adjustment formula is expressed as:


(11) $$\theta_{NN}^{\left(m+1\right)}=Adapt(\theta_{NN}^{\left(m\right)},r_{water},r_{production},\ldots )$$

In the equation, *Adapt(…)* denotes the adaptive adjustment strategy.

### 2.13 DR-DPINNs optimization modeling

**Figure 2:**
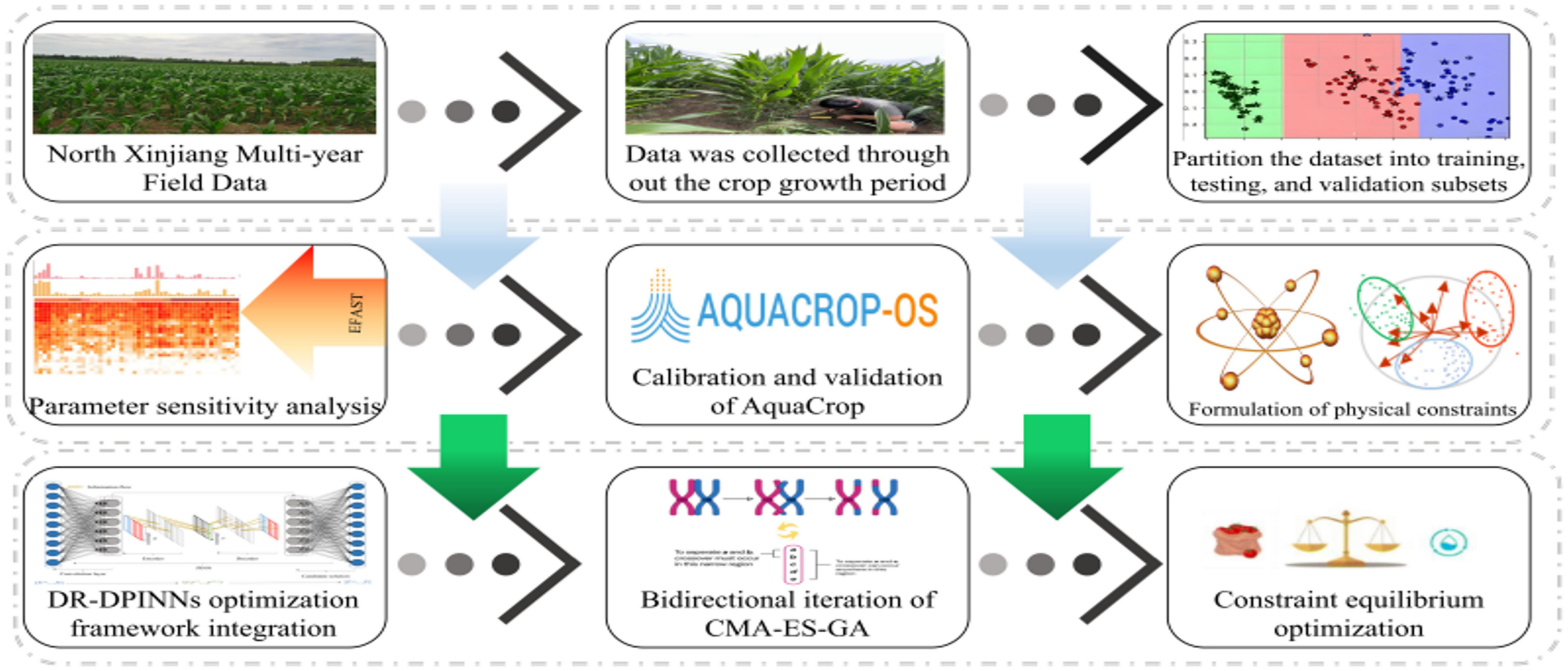
Irrigation optimization process flowchart.

In the DR-DPINNs model, the loss function has non-differentiable constraints, making traditional backpropagation algorithms ineffective for neural network parameter optimization. Therefore, this study uses a double-layer evolutionary algorithm—Covariance Matrix Adaptive Evolutionary Strategy and Genetic Algorithm (CMA-ES-GA). CMA-ES is a stochastic gradient-free optimization method, particularly suitable for solving continuous black-box optimization problems (Nomura et al., 2025). This algorithm adjusts the search distribution by adaptively modifying the covariance matrix, effectively handling ill-conditioned and nonlinear problems (Cheng et al., 2021). The learning mechanism of CMA-ES makes it invariant to any invertible linear transformation of the search space (Li et al., 2017). In contrast, GA is an optimization algorithm inspired by biological evolution. GA evolves the population through selection, crossover, and mutation operations, suitable for parameter selection and automatic search of neural networks (Mishra and Kane, 2023). The performance of PINNs is affected by hyperparameters and structural choices. CMA-ES-GA can be used to automatically search for and optimize the architecture of PINNs, enhancing their performance in solving partial differential equations and inverse problems, and further improving the training outcomes of PINNs (Kaplarevic-Malisic et al., 2023). **Figure 2** shows the Irrigation Optimization.Process Flowchart.

## 3 Results

### 3.1 Biomass parameter sensitivity

**Figure 3:**
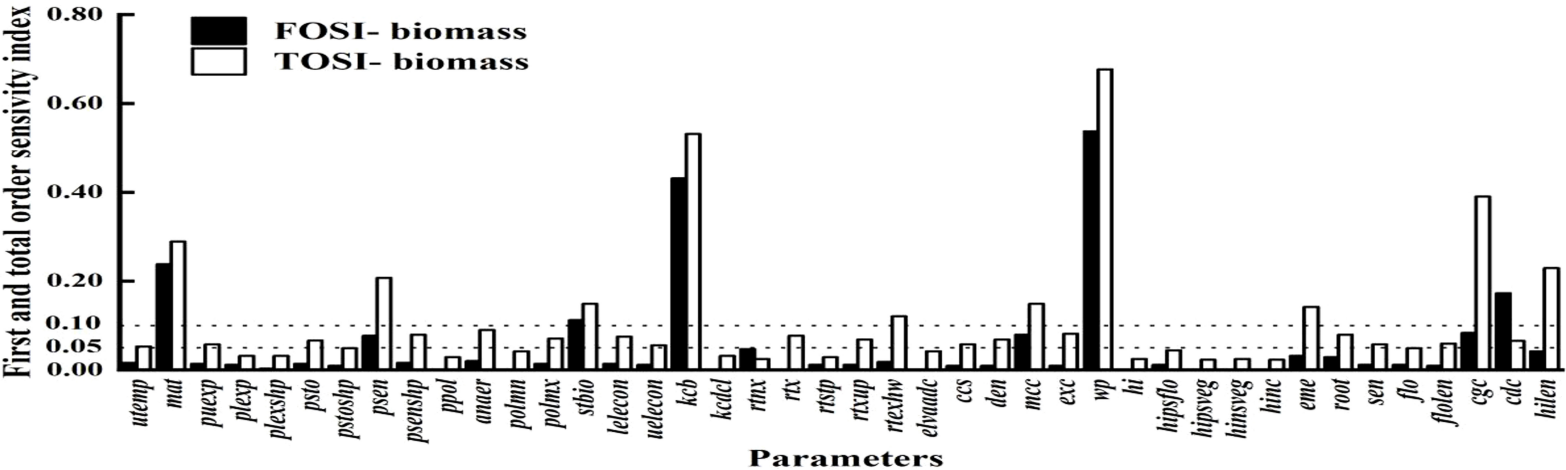
First order and global sensitivity analysis results of AquaCrop model parameters (biomass).

**Figure 3** shows the parameter sensitivity analysis for biomass. Parameters like wp, mat, kcb, stbio, cgc, mcc, psen, and cdc have Si above 0.05. Globally, STi values over 0.10 also include hilen, eme, and rtexlw. Wp stands out as a key parameter due to its significant main and interactive effects.

**Figure 4:**
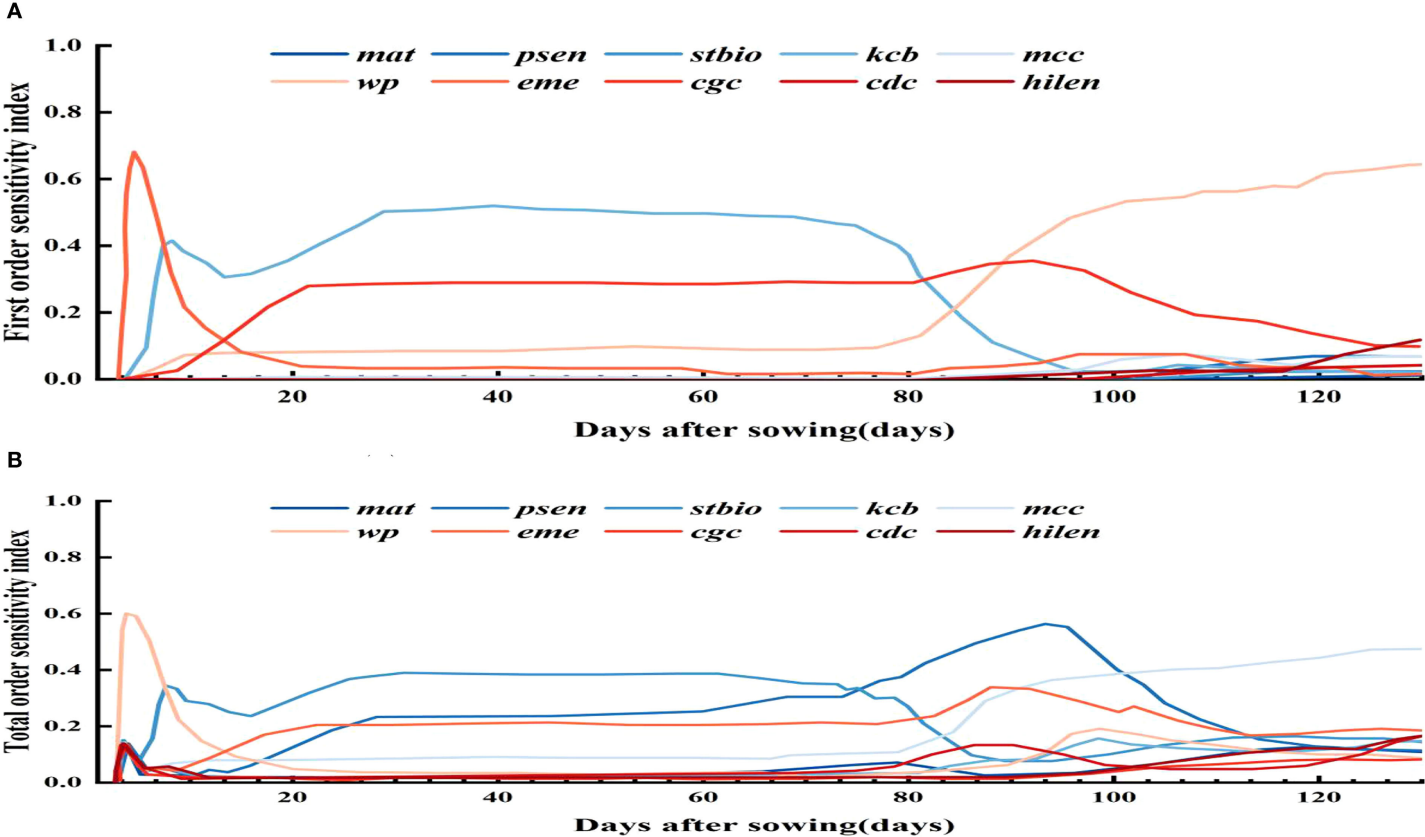
First order **(A)** and global **(B)** sensitivity analysis results of biomass over time.

To assess parameter impacts on biomass over the growth cycle, a time - varying sensitivity analysis was done. **Figure 4** highlights the top 10 parameters with Si above 0.05 and STi above 0.10, namely stbio, wp, kcb, cgc, eme, mcc, cdc, mat, psen, and hilen. Simulating their Si and STi dynamics shows stbio, wp, kcb, and cgc as the most sensitive. eme is sensitive in the early stage. stbio becomes crucial for biomass accumulation in the vigorous growth phase. kcb has high global sensitivity in the middle stage. After 80 d, wp becomes more elastic to water changes. STi rises between 90 and 120 d, indicating increased parameter interactions. cgc consistently influences biomass throughout the growth cycle.

### 3.2 Parameter sensitivity of canopy cover over time

**Figure 5:**
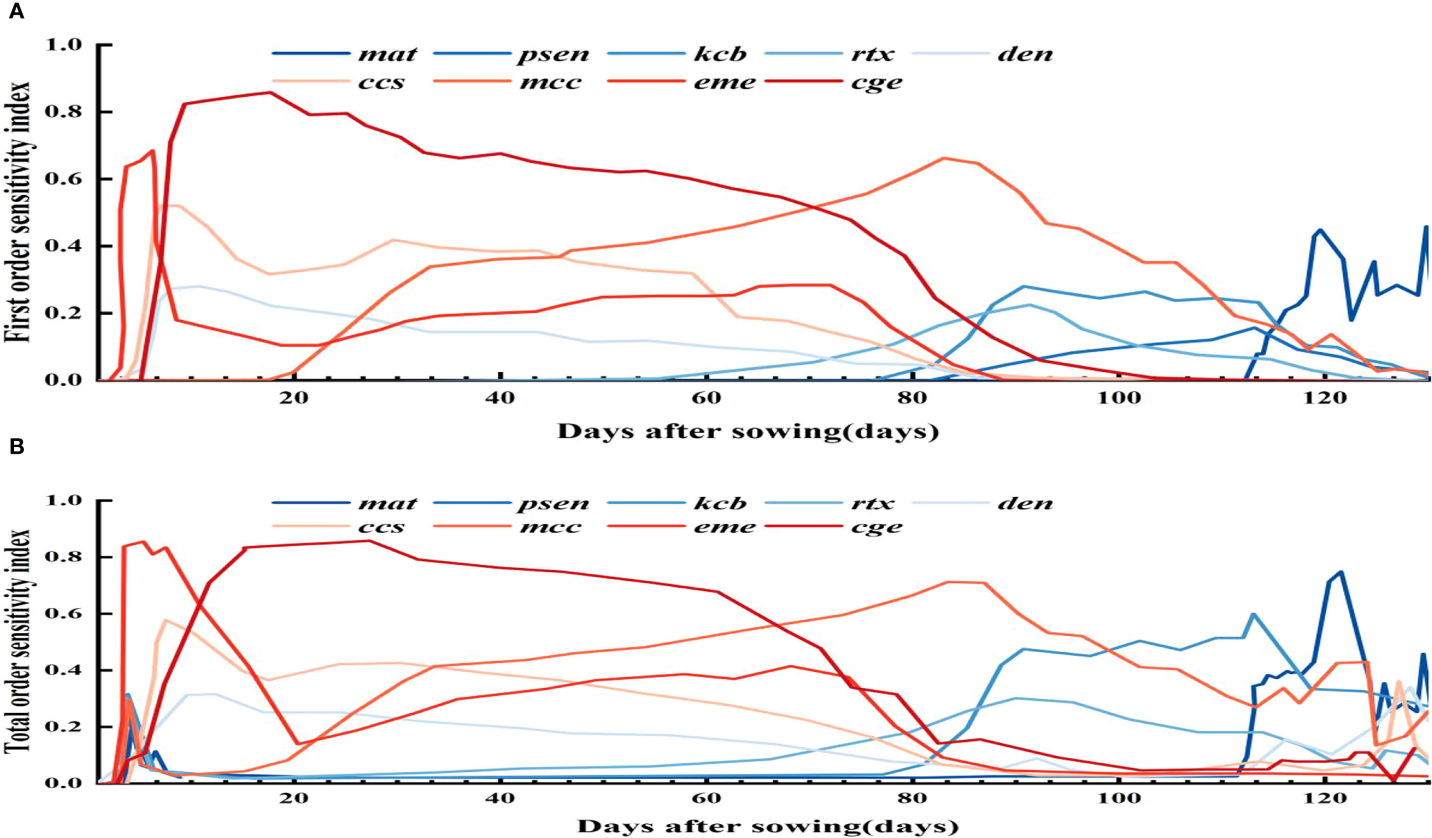
First order **(A)** and global **(B)** sensitivity analysis results of canopy cover over time.

**Figure 5** shows the time - varying parameter sensitivity analysis for canopy cover. Parameters like mcc, den, ccs, eme, kcb, rtx, mat, and psen, with Si over 0.05 and STi over 0.10, were chosen. The analysis reveals that eme and ccs have high initial sensitivity but stabilize later, indicating strong main effects on early canopy cover with limited interaction with other parameters. Between days 15 and 60, cge dictates canopy expansion, crucial for mid - season canopy closure and photosynthetic efficiency. During this period, it shows both main effects and strong interactions with other parameters. From day 60 to 100, psen’s influence on canopy senescence increases; frequent soil water deficits below its threshold accelerate canopy decline, shortening the high - cover period. In the late growth stage, most parameters affecting canopy expansion show reduced sensitivity as expansion ends and canopy enters maintenance or decline. Canopy cover development and decline depend on multiple parameters: eme, ccs, and den in the early stage; cge, mcc, and kcb in the middle stage; and mat and psen in the late decline stage.

### 3.3 Parameter sensitivity of soil water content

**Figure 6:**
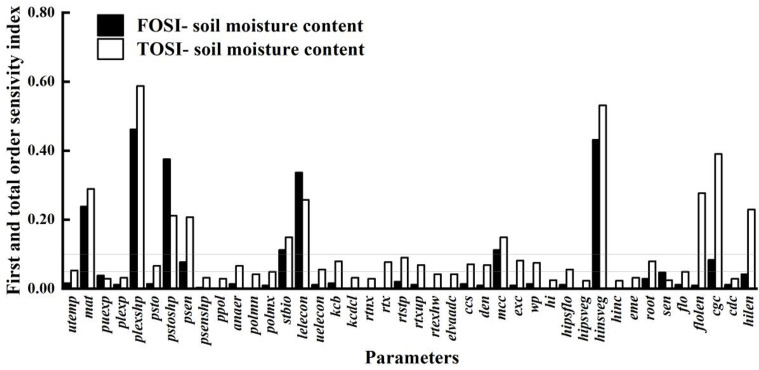
First-order and global sensitivity analysis results of AquaCrop model parameters (soil water content).

As shown in **Figure 6**, the parameter sensitivity analysis of soil water content is crucial for understanding the relationship between crop growth and water use. Compared to the sensitivity analyses of biomass and canopy cover, it shows a broader parameter impact range. This indicates that soil water content is influenced not only by parameters directly related to water dynamics but also by many indirect factors, such as crop root growth, soil physical properties, and irrigation and precipitation patterns. The crop parameters with STi above 0.10 are puexp, plexp, psen, stbio, kcb, rtx, rtexhw, mcc, wp, eme, cgc, cdc, and hilen. These parameters affect soil water content both individually and through complex interactions with other parameters, collectively determining soil moisture distribution and changes. By precisely adjusting these key parameters, soil moisture management can be enhanced, improving crop WUE and achieving water - saving and yield - increasing goals.

### 3.4 Parameter sensitivity of yield

**Figure 7:**
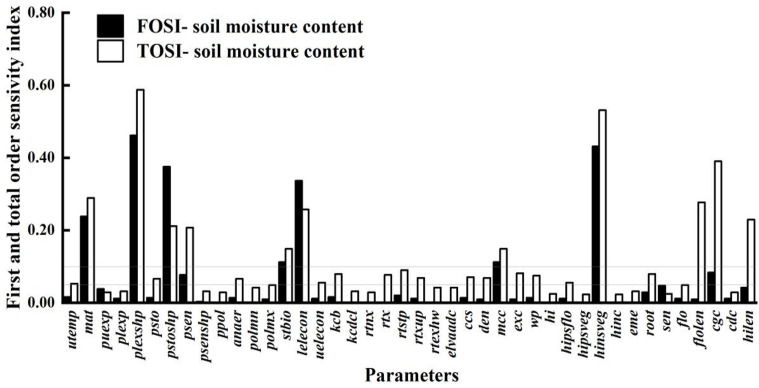
First order and global sensitivity analysis results of AquaCrop model parameters (yield).

As shown in **Figure 7**, the parameter sensitivity analysis of yield reveals a broader parameter impact range compared to biomass and canopy cover. Some parameters that only significantly affect biomass in the late growth stage also stand out in yield sensitivity. The crop parameters with a first-order sensitivity index (Si) above 0.05 include flo, psen, mat, hi, kcb, wp, and cdc. Those with a global sensitivity index (STi) above 0.10 further add hilen, flolen, hinc, sen, psenshp, and elvadc. Stress thresholds like wp, psto, and psen remain highly sensitive in yield simulation and are vital for optimizing irrigation schedules and model calibration to achieve high yield.

### 3.5 Interactions among the sensitive parameters of biomass, canopy cover, and soil water

**Figure 8:**
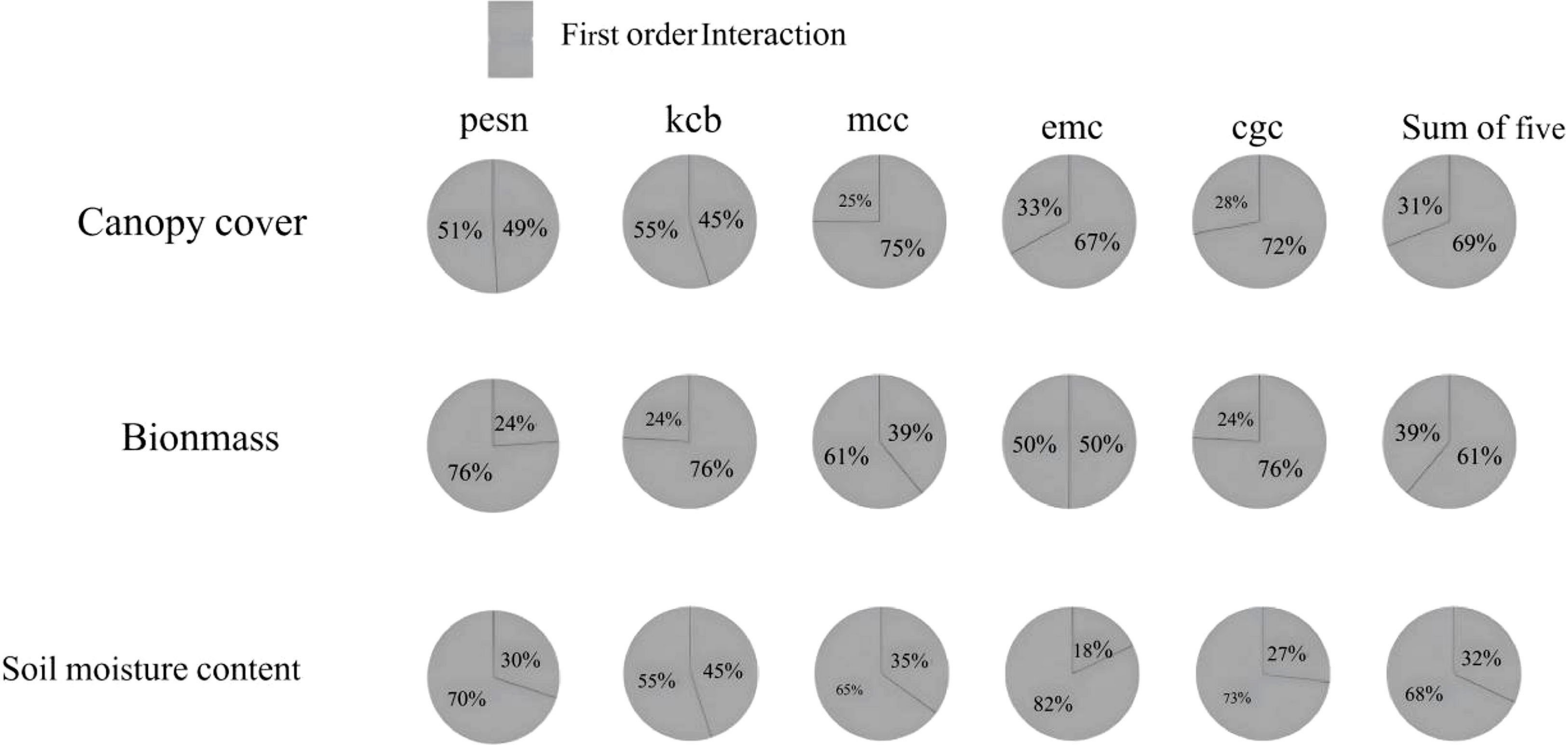
First order and interaction sensitivity index contribution rates of commonly sensitive parameters for biomass, canopy cover, and soil moisture content.

The interaction analysis of parameters affecting biomass, canopy cover, and soil moisture is shown in **Figure 8**. Biomass has significantly more determining parameters than canopy cover and soil moisture. For the same parameter, the contribution patterns to these three variables differ. Parameters directly related to canopy expansion and transpiration have a more significant first-order effect on canopy cover simulation. In biomass simulation, their interaction effects increase, indicating coupling with factors like WUE, root water uptake, and senescence rate. The contributions of key parameters to canopy cover, biomass, and soil moisture involve both strong first-order dominance and significant interaction differences. The interaction effects between key parameters and others account for a large proportion of the total contribution of jointly sensitive parameters. This indicates that the formation of canopy cover results from the synergistic effects of multiple factors. For biomass, although interaction effects are greater than first-order effects, the proportion is relatively lower than that for canopy cover. This suggests that precisely calibrating or managing the first-order effects of key parameters can significantly regulate biomass accumulation. Soil moisture is influenced by multiple factors such as precipitation, evaporation, and crop water consumption. Its sensitivity shows both first-order responses and interactions with other processes through effects on crop growth and transpiration. Therefore, priority should be given to calibrating and observing parameters with significant first-order contributions, while parameters with prominent interaction contributions require systematic optimization in combination with related factors.

### 3.6 Parameter selection and parameter calibration

The integrated sensitivity analysis lists the crop parameters in the AquaCrop model that are sensitive to maize biomass, canopy cover, and yield in the Northern Xinjiang region across different growth stages. During model localization, preset fixed values for insensitive parameters and only calibrate parameters sensitive to output variables. The parameter calibration is shown in **Table 3**.

**Table 3:** Calibration of AquaCrop model parameters.

Parameter name	Parameter description	Unit	Calibrated value
mat	Thermal Time from Sowing to Maturity	GDD	2521
eme	Thermal Time from Sowing to Emergence	GDD	151
sen	Thermal Time from Sowing to Start of Senescence	GDD	1822
flo	Thermal Time from Sowing to Flowering	GDD	1607
flolen	Thermal Time for Flowering Duration	GDD	270
cgc	Canopy Growth Coefficient	Frac GDD^-1^	0.006
cdc	Canopy Senescence Coefficient	Frac GDD^-1^	0.004
hilen	Duration for Establishing Harvest Index During Yield Formation	GDD	900
stbio	Minimum Thermal Time Requirement for Biomass Formation	GDD	12
root	Thermal Time to Reach Maximum Root Depth	GDD	1516
rinx	Minimum Effective Root Depth	m	0.3
rtx	Maximum Effective Root Depth	m	2.05
rtexup	Maximum Water Extraction in Top 1/4 of Root Zone	m^3^·(m^3^soil)^-1^·d^-1^	0.025
rtexhw	Maximum Water Extraction in Bottom 1/4 of Root Zone	m^2^·(m^2^soil)^-1^·d^-1^	0.007
rtshp	Shape Factor for Root Zone Expansion	–	15
kcb	Crop Coefficient at Full Cover but Not Senescent	–	1.1
kcdcl	Coefficient for Crop Coefficient Reduction Due to Senescence Deficit	–	0.3
evladc	Coefficient for Canopy Suppression of Soil Evaporation in Late Growing Season	–	50
wp	Normalized Water Productivity	g·m^-2^	39.7
h	Reference Harvest Index	%	49
exc	Potential Excess Fruit	%	100
puexp	Upper Soil Water Depletion Threshold Limiting Canopy Expansion	fraction TAW	0.2
plexp	Lower Soil Water Depletion Threshold Limiting Canopy Expansion	fraction TAW	0.65
pexshp	Shape Factor for Water Stress on Canopy Expansion	–	3
psto	Upper Soil Water Depletion Threshold Limiting Stomatal Conductance	fraction TAW	0.65
pstoshp	Shape Factor for Stomatal Conductance Water Stress	–	2.5
psen	Upper Soil Water Depletion Threshold Inducing Premature Canopy Senescence	fraction TAW	0.5
psenshp	Shape Factor for Premature Canopy Senescence Due to Water Stress	–	3
ppol	Upper Soil Water Depletion Threshold Limiting Pollination	fraction TAW	0.8
anaer	Anaerobic Point	%	5
polmn	Minimum Temperature Threshold for Pollination	°C	5
polmx	Maximum Temperature Threshold for Pollination	°C	35
hinsveg	Coefficient for Negative Impact on Harvest Index When Crop Growth is Limited	–	4
hinc	Maximum Allowed Increase in Harvest Index	%	15
hipsflo	Positive Impact of Water Stress on Harvest Index Before Flowering	%	3
uelecon	Lower Soil Salinity Conductivity Threshold	dS·m^-1^	5
elecon	Upper Soil Salinity Conductivity Threshold	dS·m^-1^	20
utemp	Upper Temperature Threshold for Crop Development	°C	30
den	Plant Density	plants·ha^-1^	250516
mcc	Maximum Canopy Cover	%	96.7
ccs	Area Covered per Plant at 90% Emergence	cm²	1.5
cge	Canopy Expansion Rate Coefficient	Frac GDD^-1^	0.0062

### 3.7 Simulation and validation of maize canopy cover at experimental stations

**Figure 9:**
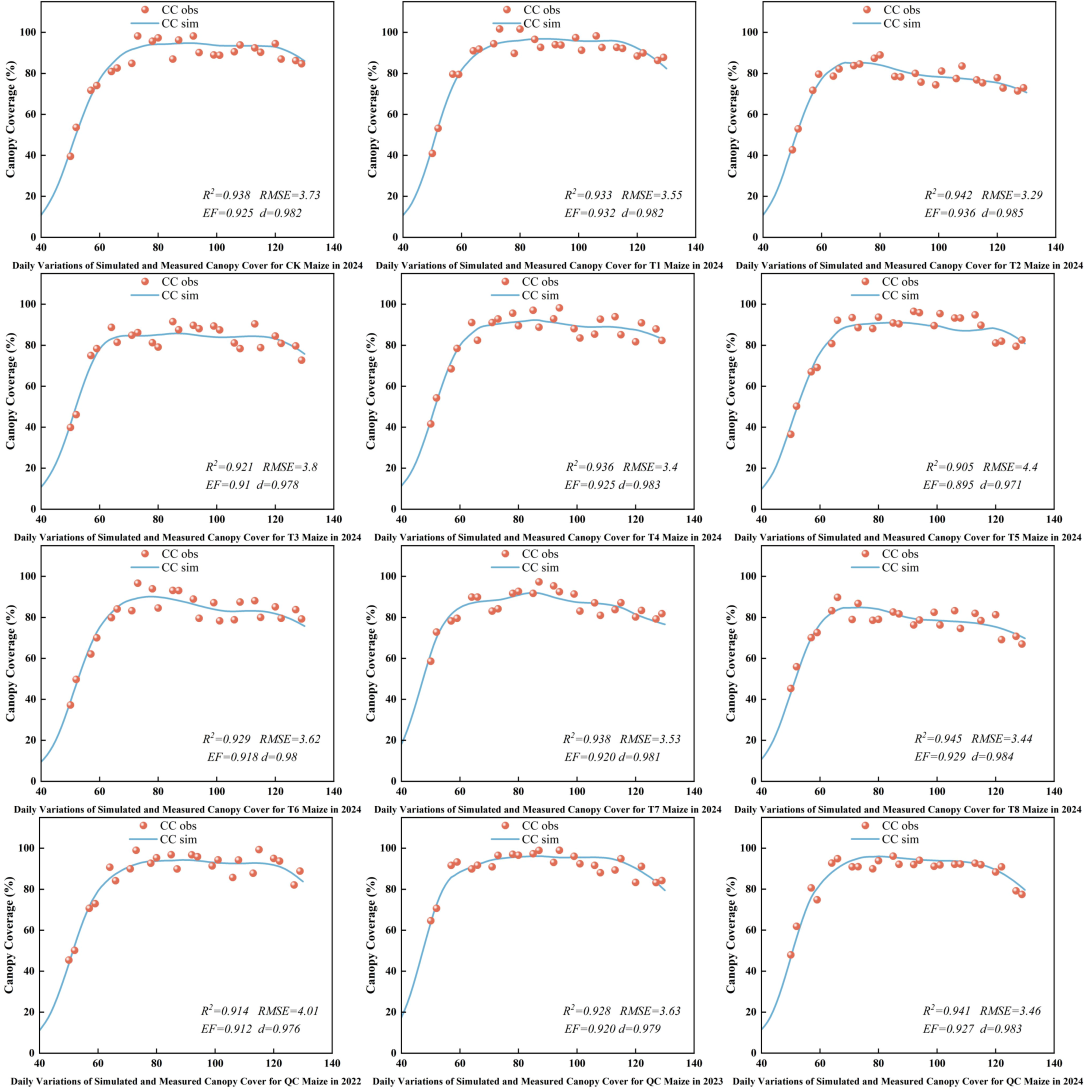
Simulation of maize canopy cover.

**Figure 9** shows the simulated canopy cover under different treatments and provides farmer practice validation results. The simulated and observed canopy cover trends were generally consistent across the maize growth period, especially under water - deficient conditions, indicating the model maintained good accuracy. The model also showed good predictive ability under farmer practice conditions, with a strong correlation between simulated and observed data.From 2022 to 2024, the R², RMSE, EF, and d values for simulated and actual maize canopy cover ranged from 0.905 to 0.945, 3.29% to 4.40%, 0.895 to 0.936, and 0.971 to 0.985, respectively. AquaCrop simulations showed good accuracy and reliability under various irrigation treatments and across different years, with high fitness to dynamic canopy cover changes.

### 3.8 Simulation and validation of maize biomass at experimental stations

**Figure 10:**
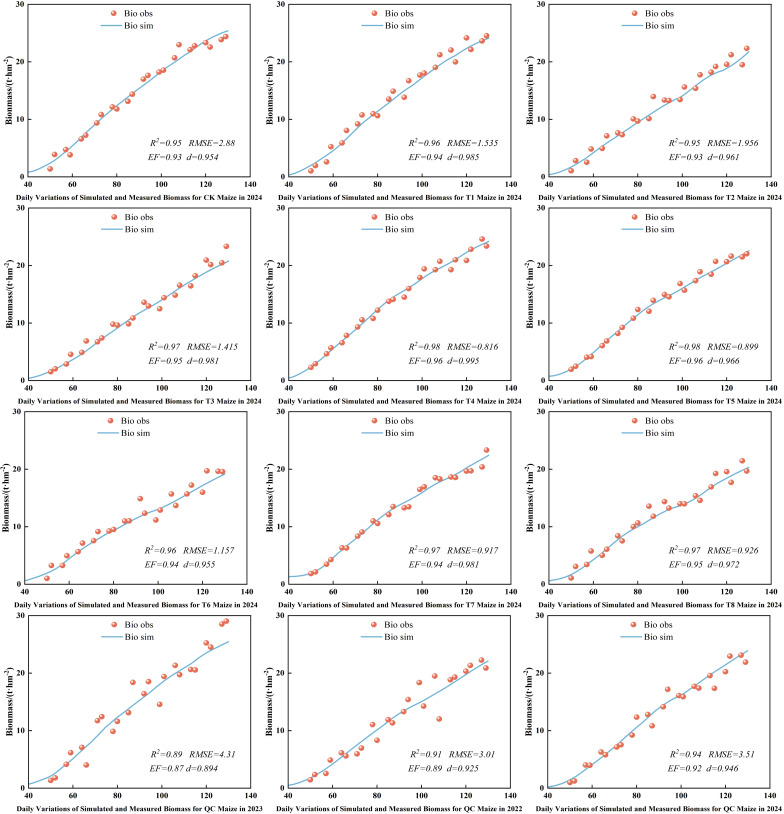
Simulation of maize aboveground biomass.

Biomass, a key indicator of crop growth and yield, was simulated under different deficit irrigation schedules to enhance maize WUE. **Figure 10** presents the simulation results and the farmer practice validation. The simulated and observed biomass showed an “S” - shaped increase over days after sowing, with the model curve closely matching the observed data. The model also accurately simulated biomass trends under various irrigation treatments and farmer practice, with high fitness between simulated and observed values. The calibrated AquaCrop model demonstrated excellent performance in simulating above - ground biomass, with R², RMSE, EF, and d values ranging from 0.89 to 0.98, 0.816 to 4.31, 0.87 to 0.96, and 0.894 to 0.985, respectively. Overall, the AquaCrop model can precisely simulate dynamic biomass accumulation under different deficit irrigation and farmer practice conditions.

### 3.9 Simulation and validation of soil moisture content for maize at experimental stations

**Figure 11:**
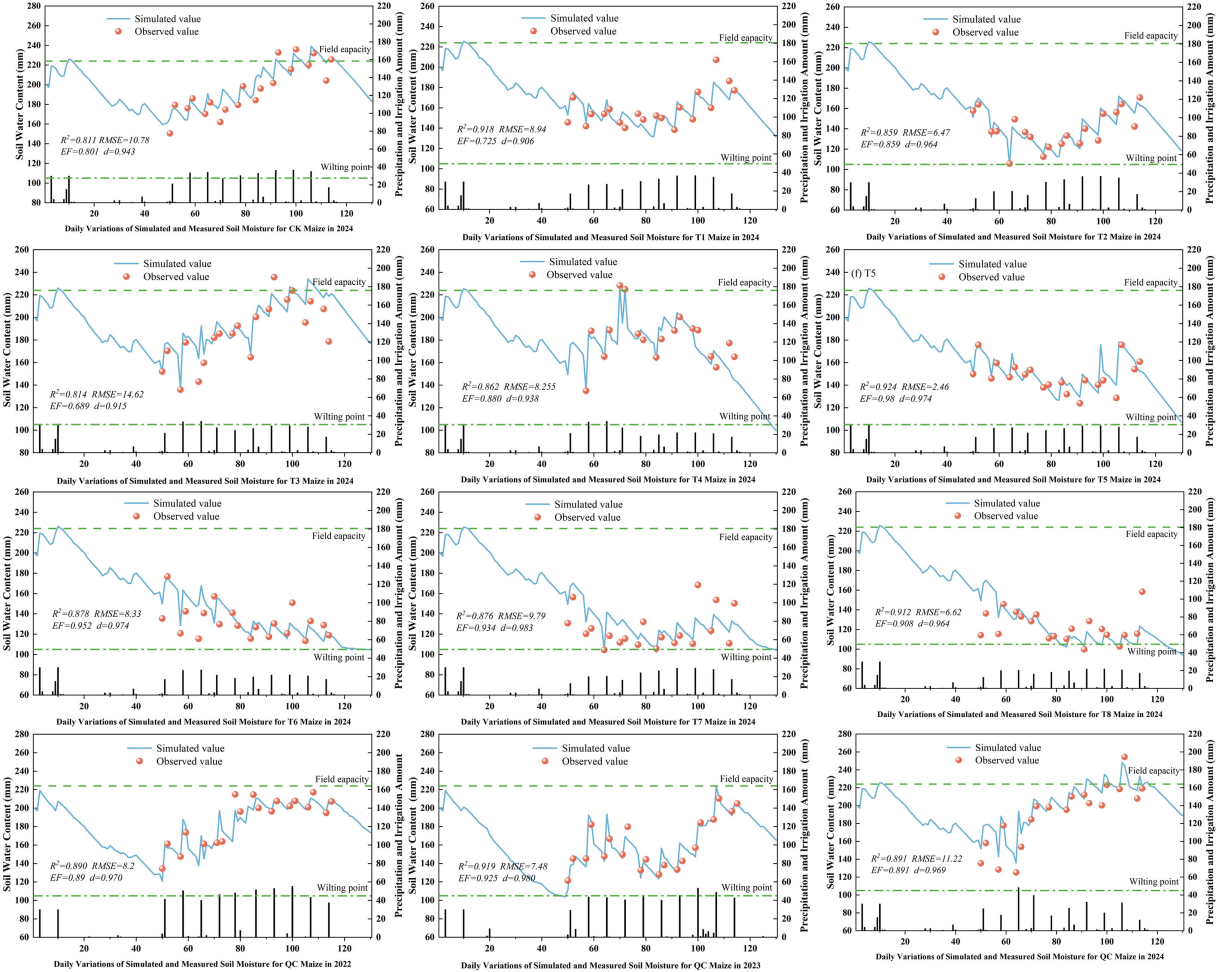
Simulation of deficit soil water content for maize.

Soil moisture, a key indicator of crop growth environment and soil health, was simulated in this study. **Figure 11** present the dynamics and discrepancies between simulated and observed soil moisture content. Under various treatments, the observed soil moisture content showed specific trends over time, closely tied to the crop growth cycle and irrigation strategies. The model - simulated soil moisture curves aligned well with observed data points, indicating the model’s ability to accurately capture the dynamic fluctuations of soil moisture content. The trends in soil moisture increase and decrease were closely related to irrigation amounts, evapotranspiration, and water uptake by crop roots. The calibrated AquaCrop model showed excellent performance in simulating soil moisture, with R², RMSE, EF, and d values ranging from 0.811 to 0.924, 6.47 to 14.62, 0.689 to 0.98, and 0.906 to 0.980, respectively. Overall, the AquaCrop model can accurately simulate the temporal changes in soil moisture under deficit irrigation across different irrigation systems. The model demonstrated a high degree of fit and good trend consistency with observed data, effectively reflecting the actual status of soil moisture.

### 3.10 Simulation and validation of maize yield at experimental stations

**Figure 12:**
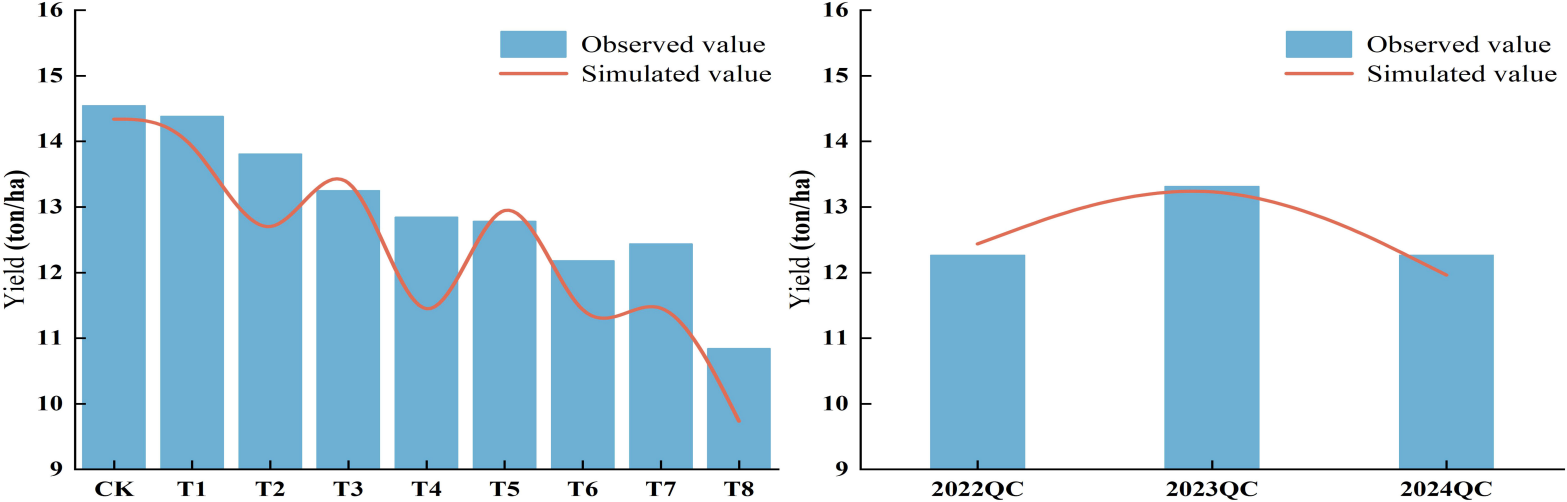
Simulation of maize yield under different treatments by the AquaCrop model.

**Figure 12** and **Table 4** shows the differences between simulated and actual yield values. The relative errors for all treatments ranged from 0.88% to 10.87%, with an average below 4.17%, indicating a good fit between simulated and observed values. The model slightly overestimated yields for T3, T5, 2022QC, and 2023QC, with overestimation ranging from 0.88% to 3.23%. In contrast, the model underestimated yields for the other treatments, with underestimation ranging from 1.42% to 10.87%. Overall, the model demonstrated high accuracy in simulating maize yield.

**Table 4:** Simulation performance of the AquaCrop model for yield under different treatments.

Treament	Observed value (kg hm^-2^)	Simulated value (kg hm^-2^)	RE/%
CK	14545.68	14339.73	-1.42%
T1	14383.15	13924.62	-3.19%
T2	13807.71	12703.77	-8.00%
T3	13251.24	13367.36	0.88%
T4	12847.3	11450.82	-10.87%
T5	12782.28	12947.36	1.29%
T6	12183.15	11436.24	-6.13%
T7	12437.72	11458.47	-7.87%
T8	10843.33	9736.75	-10.21%
2022QC	12266.04	12437.94	1.40%
2023QC	13316.36	13746.3	3.23%
2024QC	12266.04	11964.37	-2.46%

### 3.11 Deficit irrigation scheduling optimization based on DR-DPINNs

After conducting sensitivity analysis and calibration of key AquaCrop model parameters, this study employed a DR-DPINNs - based model to optimize deficit irrigation scheduling for maize in Northern Xinjiang. To ensure good initial convergence and physical consistency, initial parameters were set for the physics-informed neural network. **Table 5** lists these initial parameters.

**Table 5:** Physics-informed neural network parameters.

Parameter	Physical meaning	Value
layers	Number of hidden layers	2
n	Number of neurons	17
θ	Learning rate	0.00208
epoch	Number of iterations	20000
$N_{f}$	Number of collocation points	8000
$\omega_{f}$	Weight of physical residua	1
$\omega_{d}$	Weight of data residual	500^-1^

During the optimization of deficit irrigation scheduling using DPINNs, strong spatiotemporal dependencies and nonlinear characteristics were observed. To address the problems of gradient disappearance and local convergence in the later stages of training, DR was employed. It performed dimensionality reduction and feature extraction on key parameters during network training. This allowed the network to capture spatiotemporal dynamics while minimizing redundant noise.

**Figure 13:**
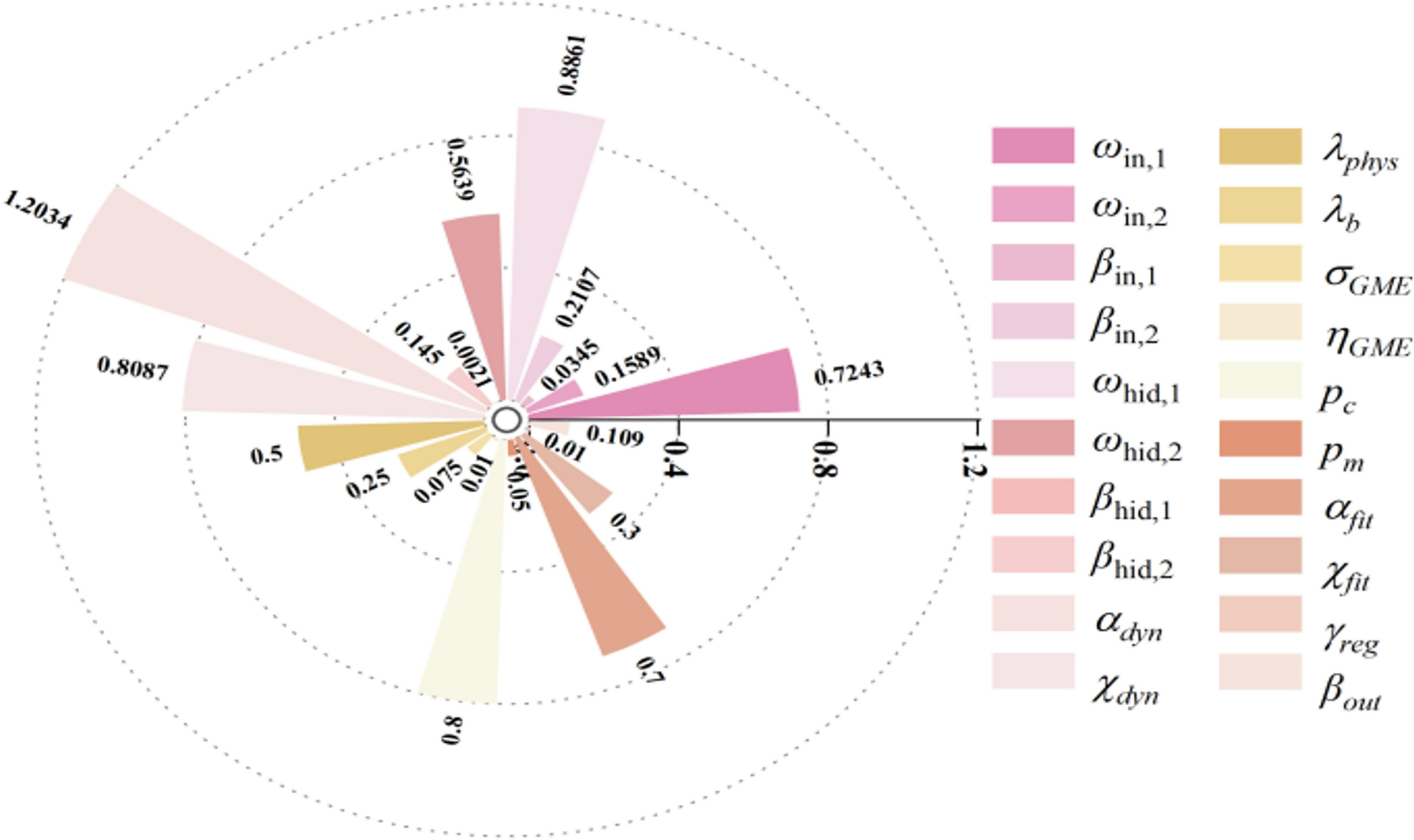
Dynamic reconstruction of the diffusion process.

On one hand, meteorological conditions, soil moisture, and crop water consumption are highly nonlinear and fluctuate significantly over time. On the other hand, traditional static networks often fail to adequately capture the complex water stress effects in the mid - to late - stages of training. To tackle these issues, DR - DPINNs incorporates a universal feature mapping layer and conducts dimensionality reduction and multi - scale feature extraction on high - dimensional inputs. When the partial differential constraint between soil water balance and crop water production functions fails to converge, an adaptive diffusion process is activated. This enables updates to certain weights and activation function parameters within a larger search space, diffusing the network’s response patterns across different growth stages (as shown in **Figure 13**). If the constraint residual remains high after several iterations, the system increases the weight of the corresponding physical residual and performs random diffusion on parameters in the output and intermediate layers. This strategy allows the network to flexibly allocate feature information across stages, effectively reducing overfitting and pseudo - convergence in DPINNs when dealing with complex spatiotemporal data.

**Figure 14:**
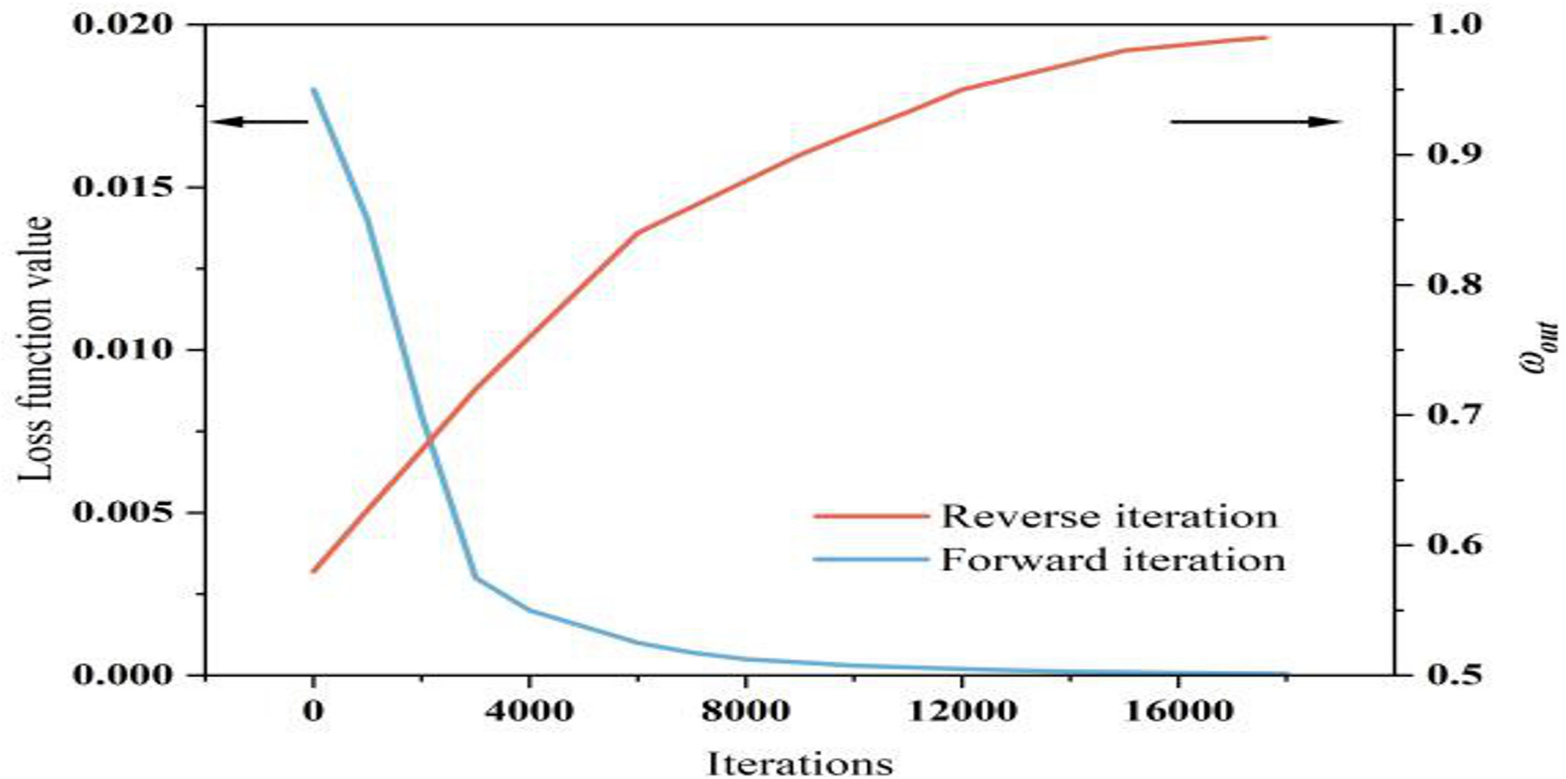
Optimization process of DR-DPINNs.

The loss function of DPINNs includes differentiable and non - differentiable constraints, and the temporal decision variables of irrigation scheduling are essentially discrete, making traditional gradient descent unsuitable. This study adopted a CMA - ES - GA double - layer iteration. In the inner network(as shown in **Figure 14**), CMA - ES performs adaptive evolution of network weights, biases, and activation function parameters under DR. CMA - ES updates the network structure globally by adaptively adjusting the covariance matrix of the initial population, reducing the deviation between soil water balance and crop water production functions. After 17, 600 iterations, the network weights stabilized, and physical constraints and observed data achieved a good fit. In the outer network, GA optimizes the irrigation decision variables globally to enhance yield and WUE. Given a fixed total irrigation quota, GA efficiently searches for the optimal irrigation allocation across growth stages. After 18, 000 iterations, the loss residual stabilized and fitness plateaued, indicating convergence of parameter updates and irrigation solutions.

**Figure 15:**
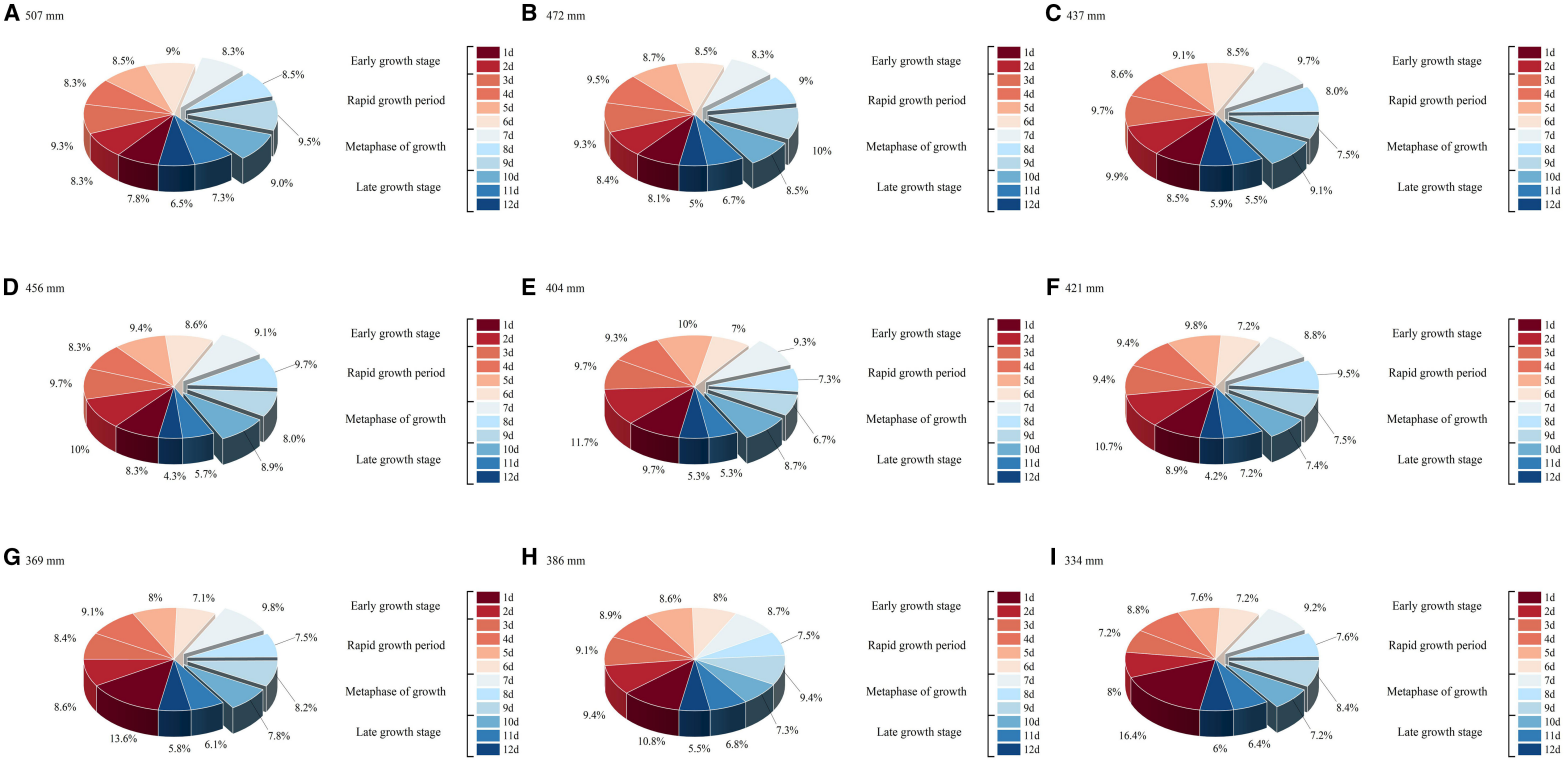
Optimization allocation results of DR-DPINNs. **(A–I)** in the figure correspond to the irrigation amounts of treatments CK–T8. Each pie chart illustrates the distribution of irrigation allocations under DR-DPINNs optimization across different growth stages: two irrigations during the early growth stage, four during the rapid growth period, three during the metaphase of growth, and three during the late growth stage. The total of 12 irrigations spanning days 1–12 is represented by percentages shown in the pie charts, indicating the proportion of total irrigation applied at each event.

To validate the DR and DPINNs method for optimizing deficit irrigation in drip - irrigated maize in Northern Xinjiang, nine irrigation - amount scenarios were tested as optimization schemes, each discretizing irrigation scheduling into four growth stages (A total of twelve irrigation events were carried out during the entire growth period, specifically on days 1 to 12.) for water allocation. **Figure 15** presents the optimal irrigation distribution results from DPINNs, considering crop water sensitivity and soil - water balance. When water was relatively abundant, more was allocated to the rapid - growth and mid - growth stages to avoid critical - stage water stress. Under water scarcity, some irrigation was shifted forward to meet seedling and early - rapid - growth - stage demands, minimizing late - stage irreversible growth losses.

The optimal irrigation schedule from **Figure 15** was used in the AquaCrop model to simulate yield, with results compared to a conventional unoptimized schedule and shown in **Table 6**. DR-DPINNs optimized the irrigation distribution to match maize water requirements, enhancing yield under all tested irrigation volumes. Yields increased by 8.35% to 10.08%, with the most significant boost at the 472 mm irrigation volume — from 13, 924.62 kg hm^-2^ to 15, 328.70 kg hm^-2^, a 10.08% rise. This mainly occurred because DR increased the model’s physical constraint weight in the jointing to tasseling stages, ensuring efficient dry matter accumulation during rapid root water uptake and high grain-filling water sensitivity. Even at the higher 507 mm irrigation volume, yields rose by 9.15%, indicating that DR-DPINNs fine-tuned irrigation for each growth stage to unlock more crop productivity. However, yield gains were more limited at lower irrigation volumes, as severe water deficits required prioritizing water for early growth stages to ensure seedling development, with mid-to-late-stage water stress being harder to offset. Still, DR-DPINNs optimized the irrigation sequence across growth stages to varying degrees, delivering yield improvements across the board.

**Table 6:** Comparison of optimized and unoptimized yields and WUE.

Irrigation treatment (mm)	Corresponding plan	Unoptimized yield (kg hm^-2^)	Optimized yield (kg hm^-2^)	Unoptimized WUE (kg hm^-2^ mm^-1^)	Optimized WUE (kg hm^-2^ mm^-1^)
507	CK	14339.73	15651.27	28.99	31.34
472	T1	13924.62	15328.70	30.47	33.87
437	T2	12703.77	13982.48	29.32	31.69
456	T3	13367.36	14608.87	27.59	30.30
404	T4	11450.82	12566.50	28.52	30.23
421	T5	12947.36	14182.96	25.85	28.19
369	T6	11436.24	12564.25	25.22	26.72
386	T7	11458.47	12586.35	26.62	28.74
334	T8	9736.75	10550.24	25.51	27.09

The optimized irrigation strategy also significantly improved WUE. At high irrigation levels of 472 mm and 507 mm, WUE increased from 28.99 and 30.47 to 31.34 and 33.87 kg hm^−2^ mm^−1^, respectively, with a maximum increase of 11.15%. At low irrigation levels of 334 mm and 369 mm, WUE still showed slight improvements, indicating that optimized scheduling can enhance water efficiency even when water is scarce. Most notably, at a total irrigation volume of 472 mm, WUE rose from 30.47 to 33.87 kg hm^−2^ mm^−1^, achieving the highest growth rate. In summary, DR-DPINNs performed well across all irrigation levels. At high levels, it better met maize growth needs, boosting yield and WUE. At medium levels, it improved both yield and WUE through rational water distribution. At low levels, it significantly enhanced yield and WUE by efficiently allocating limited water. DR-DPINNs dynamically adjusts irrigation allocation based on maize water requirements at different growth stages. At 472 mm, it achieved the best balance between maintaining high yield and improving WUE. At 507 mm, while yield increased further, the rise in WUE was smaller, suggesting that moderate deficit irrigation can yield more significant water-saving and production-enhancing effects when water is abundant.

## 4 Discussion

Crop model parameters interact significantly with regional environments. This study used global sensitivity analysis to identify key parameters affecting maize biomass, canopy cover, soil moisture, and yield, and then calibrated and validated the model using local data. AquaCrop is a model with high simulation accuracy and wide application. But its parameters must be locally calibrated. Due to differences in climate, soil, and crop varieties across regions, this calibration is needed to enhance the model’s applicability and accuracy in specific areas (Montoya et al., 2016; Coudron et al., 2023). Calibration ensures the model accurately reflects local crop growth and yield mechanisms, providing reliable support for agricultural management decisions (Akbari E. et al., 2024a; Cao et al., 2024; Han et al., 2020). Global sensitivity analysis can identify parameters with the greatest impact on model outputs. These parameters need more attention in model calibration and optimization, as their slight changes can significantly affect the model’s results. By pinpointing these key parameters, resources and efforts can be better allocated, improving the efficiency and accuracy of model calibration (Lu et al., 2021). In this study, global sensitivity analysis revealed that water productivity (wp), canopy growth coefficient (cge), and canopy senescence threshold (psen) are critical for biomass accumulation and yield. The sensitivity of wp increases in the late growth stage, reflecting the lagged impact of water stress on photosynthetic assimilate allocation. In contrast, cge dominates canopy expansion in the middle and early stages, with its interactive effects peaking during the rapid growth phase. This aligns with the findings of Jin et al. (2016) and Nasrolahi et al. (2024). Psen significantly affects yield (Si>0.05) by regulating the leaf senescence rate and the duration of grain filling. Root parameters are highly sensitive to soil moisture dynamics, underscoring the importance of optimizing root zone water extraction for water - saving and yield enhancement. However, the sensitivity of rtx and rtexkw to soil moisture dynamics was higher than expected, contradicting Akbari E. et al. (2024b), who found root parameters to be less important in a global sensitivity analysis of silage maize AquaCrop parameters. This discrepancy may stem from the unique root spatial distribution and water extraction patterns of different maize varieties under drip irrigation, and in - situ root zone observations are needed for further validation.

Simulations with the calibrated AquaCrop model show high accuracy in simulating maize canopy cover, above - ground biomass, and yield under various treatments. The model aligns well with water supply levels across growth stages, effectively capturing crop growth dynamics even under water - deficient conditions. This aligns with the findings of Yin et al. (2023) and Jin et al. (2020), confirming AquaCrop’s suitability in the arid Northern Xinjiang region and its potential as a reliable tool for maize water management.

However, the model’s precision in simulating soil moisture content is relatively low. This may stem from heavy rains during the maize rapid - growth stage in the experimental area, which increased water content in farmer - practice plots beyond experimental levels. Coupled with poor field management in some areas, these factors likely account for the discrepancies with the results of Akbari A. et al. (2024).

The experimental results show that maize is most sensitive to water deficits during the rapid growth stage, which is closely related to its physiological characteristics. During this stage, maize grows rapidly and has high water demands, and water deficits can directly affect physiological processes such as photosynthesis, nutrient absorption, and cell expansion (Li et al., 2024). Mild water deficits during this stage can slightly reduce canopy cover, but yield losses can be mitigated by compensation irrigation in the mid-to-late stages. This suggests that irrigation strategies can be flexibly adjusted in practice based on weather conditions and soil moisture to achieve water conservation and efficiency enhancement (Suriadi et al., 2024). Severe water deficits in the mid - growth stage can lead to irreversible dry matter losses. The reproductive growth stage is crucial for maize yield formation. Water stress can significantly affect pollen viability, pollination success, and grain development, resulting in reduced dry matter accumulation and yield declines. Adequate water supply should be ensured during the reproductive growth stage to avoid severe water deficits (Song et al., 2019). This is supported by the findings of Reemala et al. (2024) regarding the impact of water stress on the critical period of reproductive growth. The study by Kögler and Söffker (2020) using an open-loop control method shows that moderate water stress can trigger adaptive adjustments such as reduced stomatal conductance and restructured root architecture. These changes prompt the plant to prioritize resource allocation to reproductive organs, which may underpin the compensation of yield losses. Thus, in the face of future climate change challenges, moderate deficit irrigation is more conducive to maize production, which is basically consistent with the research results of Melkie et al. (2024).

The DR-DPINNs method, through DR and dual physical constraints, effectively addresses the limitations of traditional data-driven models in capturing mechanistic information in irrigation scheduling optimization and the neglect of deficit irrigation in physical-principle-based studies (Mortazavizadeh et al., 2025). DR, using multi-scale feature extraction and adaptive dimensionality reduction (**Figure 15**), maps high-dimensional nonlinear spatiotemporal data to low-dimensional feature spaces. This preserves key crop growth dynamics while significantly reducing model sensitivity to redundant noise. This mechanism is implemented through the dynamic feature mapping function *D(Z(t))* in Equation 11, enabling the network to adaptively capture the coupled effects of weather fluctuations and soil moisture dynamics throughout the crop growth period. The dual physical constraints, via dual residual terms from the soil water balance partial differential equation and crop water production function in Equation 10, integrate crop physiological mechanisms and soil hydrodynamics into the neural network training. Unlike single - physical - embedding methods, this study dynamically couples crop growth and water migration processes by discretizing the time - continuous conservation equation (Equations 7, 8), ensuring physical consistency in deficit irrigation conditions (Zheng et al., 2023). The DR-DPINNs optimization results indicate that, with a total irrigation of 472 mm, the yield increased by 10.08% and WUE improved by 11.05%. The optimized irrigation scheduling effectively eased water stress during critical growth stages. According to the CMA-ES-GA algorithm, the model dynamically adjusted the irrigation weights for each growth stage within the constraints of the total water volume. The proportion of irrigation during the rapid growth stage increased from 30.8% (in the low irrigation scheme) to 36%, while the proportion in the late growth stage decreased from 12.4% to 10%.By reallocate irrigation timing, this strategy takes full advantage of the active new root growth and high nutrient absorption efficiency during the rapid growth stage. The dry matter conversion efficiency per unit of water input is higher than in other stages (Nawaz et al., 2024), which is likely the main reason for the increase in yield. This is consistent with the findings of Comas et al. (2019). The DR optimization strategy in this study enables multi-stage, multi-objective collaborative optimization, offering a new technical approach for precision irrigation management in arid regions. By dynamically adjusting irrigation ratios across growth stages, a high WUE is maintained even under low irrigation quotas. Despite limited yield increases due to total water constraints, the adaptive diffusion mechanism in Equation 9 prioritizes water allocation to the early and late growth stages, keeping WUE at 26.72–26.09 kg·hm^-^²·mm^-^¹. This ‘protect the start and end, control the middle’ strategy aligns with the findings of Painagan and Ella (2022). on alleviating seasonal drought pressure through precise water scheduling. Notably, the model automatically limits over-irrigation during rapid growth under low quotas. This is likely due to oxygen reduction in saturated soils causing root hypoxia, inhibiting root growth and nutrient absorption, and thus impairing reproductive organ development, which is consistent with Yang’s research (Yang et al., 2023).

It should be noted that while the DR-DPINNs method performs well in irrigation optimization, it has high computational complexity and does not account for the sudden impacts of extreme weather events on crop growth. In addition, model parameter calibration relies on local experimental data. Future research should combine multi - regional and multi - year data to verify its generalization ability. It is recommended that future studies integrate climate change scenarios to simulate adaptive irrigation strategies under future water resource stresses and combine remote sensing data with crop models to improve strategies. This can achieve real - time monitoring and optimization of regional water dynamics. This approach is consistent with the recommendations of Wang et al. (2024) and can provide more comprehensive decision - making support for sustainable agricultural development.

## 5 Conclusions

In the AquaCrop model, wp, cge, and psen are key factors for biomass accumulation and yield. The eme parameter is crucial for the formation of early canopy cover, while the sen parameter significantly impacts late-stage canopy senescence and yield formation. The cgc parameter significantly affects canopy expansion and photosynthetic efficiency during the middle growth stage, while the cdc parameter plays an important role in late-stage canopy senescence and yield formation.

The model parameters obtained through sensitivity analysis are capable of meeting the application requirements for simulating biomass, canopy cover, soil water content, and yield in the AquaCrop model. After optimization with DR-DPINNs, the WUE of yield under different treatments was significantly improved. In the experimental optimal scenario with a total irrigation volume of 472 mm, the yield increased by 10.08% and WUE improved by 11.15% compared to conventional methods.

The DR-DPINNs method, by combining physical mechanisms and dynamic feature extraction, enhances the ability to solve high-dimensional nonlinear irrigation optimization problems, achieving simultaneous increases in yield and WUE. This study confirms the high reliability of AquaCrop in simulating the dynamic response of maize to water in northern Xinjiang and demonstrates that its integration with DR-DPINNs provides a theoretical method with mechanistic interpretability and decision-making precision for optimizing irrigation schedules in arid regions.

## Data Availability

The original contributions presented in the study are included in the article/supplementary material. Further inquiries can be directed to the corresponding author.
